# Selective Individual Primary Cell Capture Using Locally Bio-Functionalized Micropores

**DOI:** 10.1371/journal.pone.0057717

**Published:** 2013-03-01

**Authors:** Jie Liu, Radoslaw Bombera, Loïc Leroy, Yoann Roupioz, Dieudonné R. Baganizi, Patrice N. Marche, Vincent Haguet, Pascal Mailley, Thierry Livache

**Affiliations:** 1 Institut Nanosciences et Cryogénie, UMR5819 CEA/CNRS/UJF, Grenoble, France; 2 Institut Albert Bonniot, U823 INSERM/UJF, La Tronche, France; 3 Institut de Recherches en Technologies et Sciences pour le Vivant, U1038 CEA/Inserm/UJF, Grenoble, France; Northeastern University, United States of America

## Abstract

**Background:**

Solid-state micropores have been widely employed for 6 decades to recognize and size flowing unlabeled cells. However, the resistive-pulse technique presents limitations when the cells to be differentiated have overlapping dimension ranges such as B and T lymphocytes. An alternative approach would be to specifically capture cells by solid-state micropores. Here, the inner wall of 15-µm pores made in 10 µm-thick silicon membranes was covered with antibodies specific to cell surface proteins of B or T lymphocytes. The selective trapping of individual unlabeled cells in a bio-functionalized micropore makes them recognizable just using optical microscopy.

**Methodology/Principal Findings:**

We locally deposited oligodeoxynucleotide (ODN) and ODN-conjugated antibody probes on the inner wall of the micropores by forming thin films of polypyrrole-ODN copolymers using contactless electro-functionalization. The trapping capabilities of the bio-functionalized micropores were validated using optical microscopy and the resistive-pulse technique by selectively capturing polystyrene microbeads coated with complementary ODN. B or T lymphocytes from a mouse splenocyte suspension were specifically immobilized on micropore walls functionalized with complementary ODN-conjugated antibodies targeting cell surface proteins.

**Conclusions/Significance:**

The results showed that locally bio-functionalized micropores can isolate target cells from a suspension during their translocation throughout the pore, including among cells of similar dimensions in complex mixtures.

## Introduction

Purification and analysis of a distinct cell type depend on the previous isolation of a particular cell subpopulation from a heterogeneous cell mixture. Cell separation methods rely on distinctive properties of the target cells, including size, density, behavior or surface charge [1]. Gradient centrifugation, centrifugal elutriation, filtration and electrophoresis are widely used to achieve selective sorting based on physical differences between the cells in suspension [1]. Another usual approach consists in inhibiting key metabolic pathways required for cell growth or survival, such as blocking DNA synthesis (*e.g.* with hydroxyurea) or serum deprivation for a specific amount of time, to arrest the cell cycle at a particular stage, possibly eliminating unwanted cells [2].

Separation of the cells according to surface markers is of particular interest to provide highly purified populations, especially via immunolabeling of a cluster of differentiation (CD) with a fluorophore or a magnetic bead for Fluorescent Activated Cell Sorting (FACS) [3] and magnetic separation [4], respectively. Specific isolation of the cells of interest using antibodies immobilized on a solid surface has been exploited in Cell-Affinity Chromatography (CAC) devices [5]–[9] and protein arrays [10]–[14]. Affinity-based cell capture performed in miniaturized devices has been recently reported, including parallel functionalized microfluidic channels [15], [16] and single microchannels containing several antibody-coated regions [17], [18], antibody-covered micropillars [19], [20] or an antibody-coated porous membrane [21]. Shear flow is commonly employed to detach cells having low affinity with the antibody-coated surface, thus enriching cell subpopulations from initially heterogeneous cell mixtures [22]–[24]. Shear stress exerted on antibody-coated solid surfaces was also used to quantify cell adhesion [25]. Additionally, individual cells were specifically arrayed in an antibody-coated microwell array for the rapid optical characterization of cellular phenotypes [26].

Cell separation approaches are commonly combined to purify the target cell type, *e.g.* specific antibody-mediated aggregation of erythrocytes around the cells to form rosettes which are then separated by centrifugation [27], [28], cell cycle arrest followed by centrifugation [2] or CAC followed by electrokinetic separation [16]. It is also standard to quantify success or failure of cell sorting using flow cytometry [3] or the resistive-pulse technique (Coulter counter) [15], [16], [29].

An extreme case of cell separation is the capture of scarce or very rare cells [30], [31], *e.g.* circulating tumor cells, fetal cells in the mother’s blood, stem cells or induced pluripotent stem cells. CAC may provide a solution to isolate some of these cells if they own a specific antigen on their membrane [15], [16], [20].

Adhered cells can be locally detached from a solid surface using confined dispense of trypsin and aspiration of the cell [32], desorption or cleavage of the linker or probe under the cell [14], [33], [34], controlled reversal of the adhesion properties of the surface [35], laser capture microdissection [26], laser-based release of a piece of the micropatterned surface [36], or laser microdissection and catapulting of a portion of the surface [37]. A retrieved cell transferred into a culture vessel may result in a monoclonal culture [36]. Furthermore, innovative methods have been developed to examine the genome sequence [38], profile gene expression (mRNA, miRNA) [38], reveal abundant cytoplasmic peptides and small molecules [39], quantify levels of cytoplasmic and membrane proteins [40], investigate ion channels [41], [42] or detect secreted proteins [43] from isolated cells at the single-cell level.

Solid-state single pores have been successfully used for fast and label-free detection and analysis of biological objects, such as cell counting [29], [44]–[46], virus characterization [47], [48] and biomolecule discrimination [49], [50]. By driving biological objects across a single pore using an external pressure or electric field, an ionic current variation can be measured during the translocation, which may provide physical information (*i.e.* diameter and length) about the object [51]. This translocation-based analysis relies on the ability to identify the targets according to their physical dimensions. However, Coulter counter-like recognition of targets can be challenging when their size distributions are similar, as it is typically the case for cells. As an example, fluorescent labeling of B and T lymphocytes is usually required to differentiate them, as a result of their similar dimension ranges [52], [53]. Moreover, B and T lymphocytes can not be distinguished using optical microscopy because of their comparable optical properties in absence of labeling [52], [53].

The identification of biomolecules with similar size distribution was recently made possible using chemically-modified biological or solid-state nanopores [54]. The functionalization of the nanopore wall with biochemical probes increases the detection selectivity by establishing specific interactions between the nanopore surface and the targeted objects during their passage through the pore.

In this article, we demonstrate the discrimination of living cells among a heterogeneous cell population using antibody-functionalized micropores. The micropore walls were, in a first step, locally functionalized by oligodeoxynucleotide (ODN) stickers using the recently developed “Contactless Electro-Functionalization” (CLEF) technique [55]–[57]. CLEF provides chemical functionalization of the inner wall of solid-state pores with no side-effect deposition onto the bulk membranes. Detection sensitivity of flowing targets is thus enhanced by focusing target capture events only inside the micropores, *i.e.* with no missed detection events on the bulk membranes [55], [56]. Cell surface protein-specific antibodies conjugated with complementary ODNs were then immobilized on the pore walls via hybridization. The functionalized micropores were devoted to cell-type selective capture of primary living cells from a native biological sample. The translocation and capture process of B and T lymphocytes in the micropores were monitored in real time by optical microscopy.

## Results and Discussion

**Figure 1:**
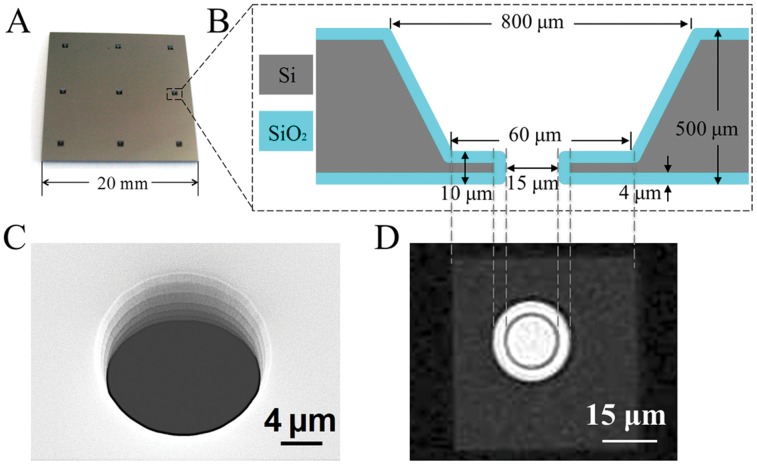
Silicon micropore chip. **A.** A photograph of the silicon micropore chip. **B.** Cross-section diagram of the pyramidal opening and the micropore in the silicon chip. A thermally grown silica layer covers the entire chip surface and the pore wall. **C.** Scanning electron microscopy image of the micropore. **D.** Optical transmission microscopy image of a micropore.

Silicon chips containing 9 micropores of 15 µm in diameter (Figure 1A) were used in this study to validate efficiency of CLEF in the simultaneous functionalization of several micropores. Micropores with scalloped inner walls were etched in the membrane conserved at the bottom of each pyramidal opening (Figure 1). The 10 µm-thick pore walls were functionalized with ODN probes using the CLEF technique [55], [56]. In brief, an electrolyte solution containing pyrrole and pyrrole-ODN monomers was filled into a reacting chamber, which was separated in two compartments by the silicon micropore chip. The number of micropores in contact with the electrolyte is adjustable from 1 to 9 depending on the dimension of the reacting chamber. Two platinum electrodes were placed in each compartment at a distance of about 3 mm from the chip surface. By applying a potential difference of 2 V between the two Pt electrodes for 100 ms, thin films of polypyrrole-ODN (PPy-ODN) copolymer were locally electro-polymerized on the inner wall of micropores in contact with the electrolyte. The functionalization efficiency was verified by fluorescence microscopy upon hybridization with complementary biotinylated ODNs and coupling with streptavidin-*R*-phycoerythrin [55], [56]. The presence of fluorescence on the pore wall confirmed the local micropore functionalization by ODNs (Figure S1 in File S1).

**Figure 2:**
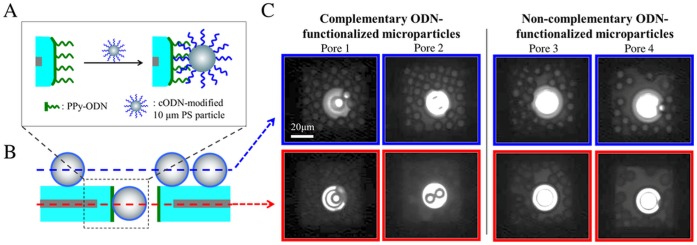
Selective capture of polystyrene (PS) microparticles in functionalized micropores. **A.** Schematic illustration of the specific interaction between ODN probe-functionalized micropore wall and the cODN target-functionalized PS particles. **B.** Schematic representation of the focusing planes for image acquisition by optical transmission microscopy. **C.** Photographs of ODN probe-modified micropores after incubation with cODN (pores 1 and 2) or ncODN (pores 3 and 4) target-functionalized particles by focusing the microscope objective on microparticles settled on the micropore membrane (blue-framed images) or inside the micropores (red-framed images).

Used as a first model, the translocation and capture experiments in functionalized micropores were assayed using ODN-modified polystyrene particles. For this purpose, PPy-ODN-functionalized micropore chips were incubated with complementary ODN-modified 10-µm polystyrene particles (PS-cODN) (Figure 2A), and observed by optical transmission microscopy. In control experiments, non-complementary ODN-modified 10-µm polystyrene particles (PS-ncODN) were used to assess non-specific microparticle adsorption. After incubation for 30 min, the micropore chips were washed in a gentle manner to remove PS-cODN or PS-ncODN adsorbed on their surface. Some microparticles remained on the chip, including on the membrane at the bottom of the pyramidal opening. Harsh wash was not employed in order to prevent detachment of the captured microparticles as high shear stress exerted on the microparticles inside the geometric restriction of the pore may peel off the pore coating and thus pull out the trapped particles. Despite the gentle washing applied, discrimination between particles remaining on the chip membranes and particles captured in functionalized micropores can be achieved by focusing observation in the pores. Using an upright microscope, two images were registered for each micropore in order to visualize the PS particles settled around or captured inside the micropores (Figure 2B). Similar high densities of settled PS particles were observed around the micropores (Figure 2C), which suggests efficient penetration of particles into each micropore during the incubation process. PS-cODN microparticles were immobilized inside the ODN-functionalized micropore, whereas no capture phenomenon was observed for PS-ncODN particles (Figure 2C).

**Figure 3:**
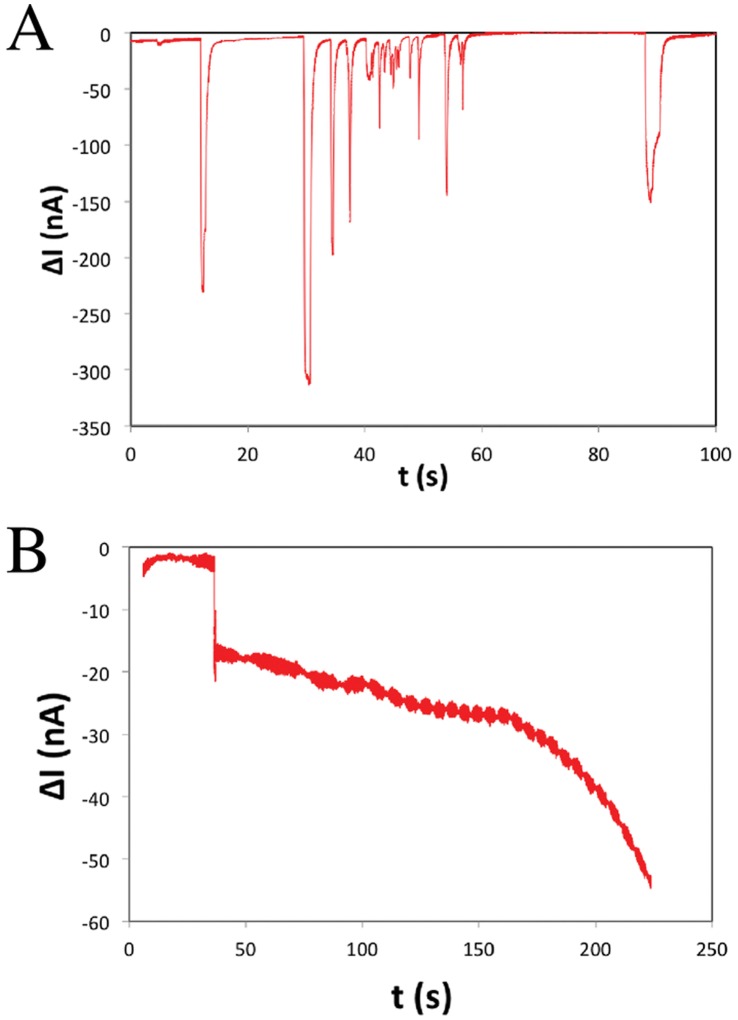
Typical current versus time traces of ODN-coated PS microparticles passing throughout an ODN-functionalized micropore. A. Translocation of PS-ncODN. B. Capture of PS-cODN. The bias potential across the micropore was 10 mV.

The dynamics of translocations of PS-cODN and PS-ncODN in ODN-functionalized micropores was investigated by recording the variation of ionic current across the micropore versus time using Ag/AgCl electrodes located few millimeters on either side of the micropore chip (Figure 3). Detection events of translocations or captures obtained by the resistive-pulse technique were far superior to the ∼1.5 nA peak-to-peak baseline noise. Typical current versus time traces for PS-ncODN displayed steady and nonblockaded translocations of these particles through the micropore (Figure 3A). The translocation duration of PS-ncODN was measured to be 1.2±0.7 s in our experiment conditions. Amplitudes of current variations ranged on two orders of magnitude, from 3.1 nA to 306 nA, likely resulting from translocation of either a single particle or small aggregates of particles. Comparable dispersion of current variation amplitudes was already noticed in our prior investigation with 100 nm-large non-complementary ODN-coated Au particles passing through 200 nm-wide ODN-functionalized solid-state pore [56].

On the opposite, PS-cODN provided permanent and cumulative blockage of the micropore as a result of PS-cODN immobilization inside the pore (Figure 3B). Similar permanent blockade of ODN-functionalized nanopore by complementary ODN-coated Au nanoparticles was also recorded in [56]. The amplitude of permanent current variation due to obstruction by PS-cODN in Figure 3B is included in the range of current variations generated by PS-ncODN translocation. The translocation and capture results illustrated in Figure 3 are in full agreement with observations in optical transmission microscopy (Figure 2) and with our similar resistive-pulse measurements [56]. We therefore conclude that the PS particle capture inside the micropore is the result of specific interaction between the locally ODN-functionalized pore wall and the cODN-modified PS particles.

**Figure 4:**
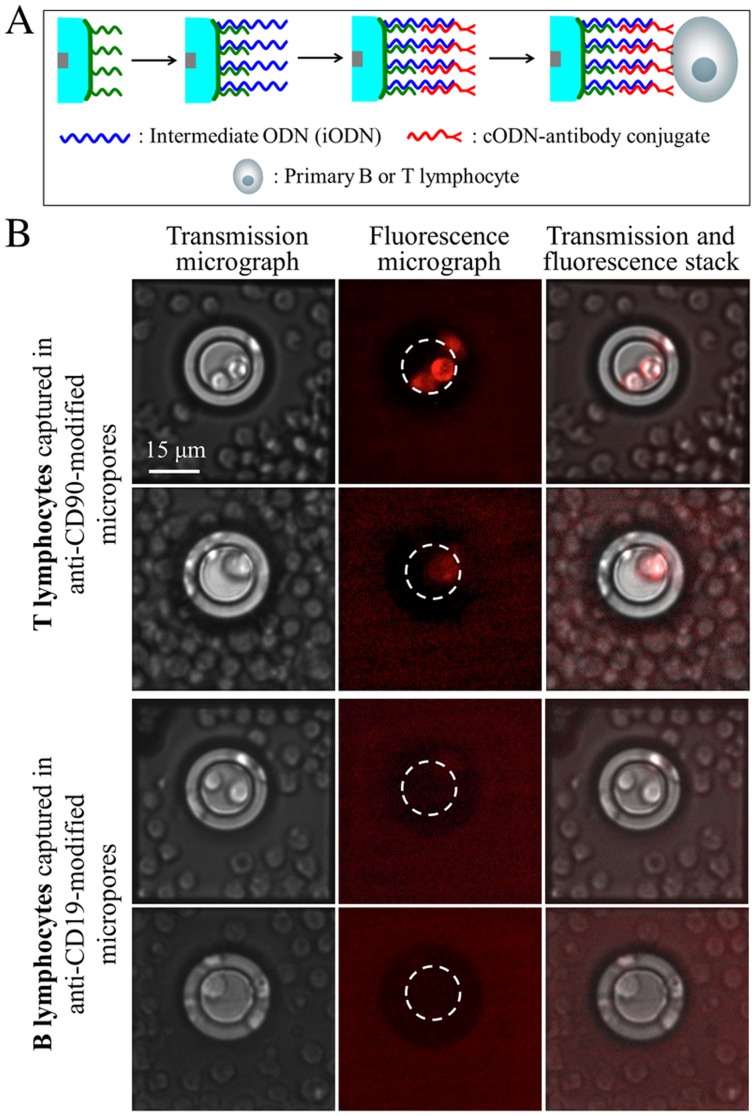
Selective capture of B or T lymphocytes from primary splenocyte samples using specific antibody-functionalized micropores. Only the T lymphocytes are fluorescently labeled. A. Schematic illustration of micropore functionalization with antibodies targeting cell surface proteins. B. Transmission and fluorescence microscopy images of cells captured in antibody-functionalized micropores, and stacks of the images. The white dashed circles in the fluorescence images indicate the position of the micropore wall.

ODN-modified micropores were converted into antibody-modified micropores by using antibody-ODN conjugates [14], [40], [58], [59]. This straightforward ODN-mediated immobilization process was used to organize the cell-specific antibodies into the different pores. To investigate the recognition capabilities of the antibody-modified micropores, mouse splenocytes containing mixed populations of B and T lymphocytes were chosen as the cellular sample because in absence of fluorescent labeling B and T lymphocytes can not be distinguished by optical microscopy as a result of their similar size ranges and optical properties [52], [53]. A sample of splenocytes was collected from the spleen of a sacrificed mouse and purified by lysis and centrifugation. FACS measurements showed that T lymphocytes and B lymphocytes accounted for 29% and 67% of the whole splenocyte population, respectively (Figure S2 in File S1). T lymphocytes were selectively labeled in the sample by fluorescent R-phycoerythrin conjugated with anti-CD3e antibody. Anti-CD19 and anti-CD90 antibodies immobilized on a surface via ODN hybridization were recently shown to be able to selectively bind to B and T cells, respectively, [14] and were thus chosen for the functionalization of micropores (Figure 4A). Prior to incubation with cells, the micropore chips were treated with bovine serum albumin (BSA) to minimize non-specific adsorption of cells on the surface and immersed in PBS buffer.

The chips were placed over an inverted microscope for *in situ* observation of the micropores (Figure S3 in File S1). 10 µL of splenocyte suspension was loaded from above the zone of each micropore. A few minutes after cell deposition, cells sedimented onto the chip membrane and entered inside the micropore. Movies S1 and S2 show cells passing within the micropores in close vicinity or in contact with the pore surface. The proximity of flowing cells with the pore surface suggests that biomolecular interactions are prone to be established between the translocating cells and antibody probes.

For antibody-functionalized micropores, two different phenomena were observed (Movie S1): individual cells either translocated through or stopped inside the pores. Epifluorescence microscopy revealed specific immobilization of T lymphocytes in anti-CD90-modified micropores, and B lymphocytes in anti-CD19-functionalized micropores (Figure 4B). In our experiment conditions, we observed that most of the antibody-functionalized pores trapped a cell among the 10 first translocating cells. No non-specific cell capture was observed in our experiments. It is worth to notice that once captured, cells remained immobilized in the micropores for the whole experiment.

ODN-modified micropores were used for control experiments to monitor the non-specific cell translocation. In absence of specific interaction, cells translocated through the 10 µm-deep micropores within 1 to 3 seconds (Movie S2). This time range is close to the translocation durations of PS-ncODN measured above using the resistive-pulse technique (1.2±0.7 s). Furthermore, no cell immobilization inside the control pores was observed (Movie S2). The above results confirm that antibody-functionalized micropores ensure selective capture of well-defined cell types from a complex mixture of cells.

In conclusion, we have demonstrated that locally functionalized micropores can be used for selective capture of individual micrometric objects. This strategy has been evidenced using cODN-modified PS microparticles, and then extended to living primary cells. B or T lymphocytes have been selectively isolated from a splenocyte suspension using micropores modified by specific antibodies. As a result of the geometric restriction of the pore, a high probability of physical contact between the flowing cells and the functionalized surface is ensured.

This approach may provide a simple way for individual cell sorting from a complex sample, by ensuring selective and spatially controlled cell capture. It has potential applications for individual cell analysis by immobilizing and optically detecting individual target cells at desired positions, then detaching them using enzymatic cleavage of a specific restriction site present in the ODN sequence, as demonstrated by some of us recently [14]. The released cells collected in the bottom compartment by sedimentation may be subject to further examination [38]–[43]. In addition, the capture of other biological objects, *e.g.* bacteria, should be possible by decreasing the pore size to that of the targeted biological objects. Possible applications of in-situ bacteria detection include fast identification and quantification of bacteria in clinical or environmental samples.

Given the possibility of one-step multi-pore functionalization, the employment of locally functionalized micropore arrays might also open up new possibilities for high-throughput sorting and collection of rare cells. High detection sensitivity may be provided by the compelled movement of the cells close to the probes in the micropores, which might be especially useful to reveal cells present at low ratio in the suspension. We therefore anticipate that the approach described herein will be useful for fundamental research in individual cell analysis as well as a detection platform for diagnostic applications. To achieve these goals, an array of bio-functionalized micropores is preferred to a single micropore approach in order to simultaneously prevent definitive blockade by only few cells, reach a high throughput of sample analysis, observe collectively the whole set of trapped cells using wide field-of-view imaging [60], [61], and possibly vary functionalization coatings and thus capture possibilities.

## Materials and Methods

### Micropore Fabrication

The micropore silicon chips were fabricated following the process described previously [42], [56]. Briefly, an array of nine 6-µm-thick 60×60-µm-large square silicon membranes were manufactured in *p*-doped silicon wafers with a resistivity of 14–22 Ω cm using backside lithography, reactive ion etching (RIE) and KOH etching. A scalloped micropore of 19 µm in diameter was then etched by deep RIE in each silicon membrane. Steam oxidation of silicon at 1050°C during about 2 days formed a 4-µm-thick silicon oxide layer on the whole chip surface, including the micropore wall, which reduced the pore diameter to 15 µm and increased the membrane thickness to 10 µm (Figure 1).

### Micropore Functionalization

The functionalization of micropores with PPy-ODN copolymer has been described previously [55], [56], and is detailed in Supporting Information (Text A and Text B in File S1). Briefly, the micropores to be specifically functionalized were placed between two compartments filled with an electrolytic solution containing pyrrole and pyrrole-ODN (Table S1 in File S1). A platinum electrode was immersed in each compartment. PPy-ODN copolymer was only electro-polymerized on the micropore inner wall upon application of 2 V for 100 ms supplied by a potentiostat SP–300 from Bio-Logic (Claix, France).

To functionalize the micropores with cell-specific antibodies, PPy-ODN-functionalized micropores were incubated with 100 nM intermediate ODN (Table S1 in File S1) in hybridization buffer (HB, 0.02 M sodium phosphate buffer, 1.1 M NaCl, 5.4 mM KCl, 4% v/v 50× Denhardt, 0.2% v/v salmon sperm DNA, 0.3% v/v Tween 20, pH = 7.4) for 15 min, and washed with washing buffer (WB, 0.01 M sodium phosphate buffer, 0.55 M NaCl, 2.7 mM KCl, 0.15% v/v Tween 20, pH = 7.4). Then the chip was incubated with 50 nM antibody-conjugated complementary ODN strands (cODN-antibody, Table S1 in File S1) in HB for 15 min to immobilize the antibodies on the pore wall and subsequently washed with WB. Two types of antibodies were used for micropore functionalization, anti-CD19 and anti-CD90, which are specific to B and T lymphocytes, respectively. Anti-CD19 and anti-CD90 immobilizations were performed on PPy-ODN sequence 1 and PPy-ODN sequence 2 functionalized micropores, respectively (Table S1 in File S1). At last, the chip was incubated in PBS containing 1% of BSA for 15 min to prevent non-specific adsorption onto the membrane surface, and immersed in PBS.

### Polystyrene Microparticle Coating and Capture in Micropore

A 0.1 mL aliquot of streptavidin-coated polystyrene (PS) microparticle suspension (1% w/v) was washed 3 times with PBS buffer and resuspended in 0.5 mL of WB. Then, biotinylated complementary or non-complementary ODN target (cODN-biot or ncODN-biot) solution (0.1 mL, 100 nM) in WB was added to the PS suspension, followed by a gentle agitation for 15 min at room temperature. At last, the PS microparticles were washed 3 times with WB and re-suspended in WB (0.5 mL). The final products were named PS-cODN and PS-ncODN, respectively.

PPy-ODN-functionalized micropores were suspended in HB with the pyramidal opening face upwards. In this configuration, non-captured microparticles driven by gravity could pass through the micropore. A PS-cODN or PS-ncODN suspension (100 µL) was added with a pipette. The translocation experiments were performed in HB buffer in order to favor capture of PS-cODN by hybridization during their passage across the micropores. Microparticles sedimented into the pyramidal openings and onto the membrane surface within few minutes. After incubation for 30 min, the chip was gently rinsed with WB and immersed in WB for observation with an upright transmission microscope (BX60 from Olympus) equipped with a chilled CCD camera (C5985 from Hamamatsu).

For resistive-pulse sensing of PS-cODN capture and PS-ncODN translocation, Ag/AgCl electrodes were plunged in the compartments filled with HB and positioned few millimeters from the micropore. A bias potential of 10 mV was applied across the micropore using the potentiostat SP-300 (Bio-Logic) equipped with shielded cables to reach nA sensitivity. The baseline current was removed to compensate asymmetry potential between the electrodes. The microparticles suspended in HB were deposited with a pipette and driven by gravity toward and throughout the ODN-functionalized micropore.

### Cell Sample Preparation and Capture in Micropore

C57BL/6 mice were purchased from Charles River. Mice were sacrificed at an age of 10 weeks by cervical dislocation. Animal housing, care and sacrifices for this study strictly followed the rules of the Animal Experiment Ethics Committee of the Université Joseph Fourier of Grenoble (Permit Number: B38 516 10 006). After the removal of the spleen, cells were separated on a grid mesh and suspended in RPMI medium. After centrifugation for 5 min at 300 *g*, the cell suspension was incubated for 5 min in the presence of a lysis buffer (8.3 g L^−1^ NH_4_Cl, 0.8 g L^−1^ NaHCO_3_, 0.04 g L^−1^ EDTA) in order to eliminate the red blood cells. After washing in PBS, B and T lymphocytes were centrifuged again (5 min, 300 *g*) and seeded at 10^6^ cells/mL in RPMI containing Fetal Bovine Serum (FBS, 10%), penicillin (50 U mL^−1^) and streptomycin (50 µg mL^−1^). The cultures were incubated at 37°C in a humidified (95%) incubator with 5% CO_2_ for 24 h in the presence of a stimulation factor (2 µg mL^−1^ Concanavalin A). Subsequently, the splenocytes were centrifuged (300 *g*, 5 min) and dispersed in PBS at 10^7^ cells/mL. The cellular sample was then incubated with *R*-Phycoerythrin-conjugated anti-CD3e IgG (15 min, 4°C, in dark) for specific immunostaining of T lymphocytes. At last, the cells were washed twice with PBS and re-suspended in PBS at 10^7^ cells/mL before use. 10 µL of splenocyte suspension was added from the top of the pyramidal opening of the functionalized micropores. Cell translocation and capture dynamics in the micropore was monitored in real time with an inverted transmission microscope (DMI 4000 B from Leica) equipped with a CCD camera (Pike F145B from Allied Vision Technologies, Stadtroda, Germany). Fluorescence microscopy of lymphocytes trapped in the micropores was performed using the epifluorescence microscope BX60 (Olympus) with the chilled CCD camera C5985 (Hamamatsu).

## Supporting Information

File S1 This file contains: **Table S1 Sequences of the probes and ODN conjugates used in this study.** Anti-CD19 and anti-CD90 are specific to B lymphocytes and T lymphocytes, respectively. **Figure S1 Characterization of PPy-ODN-functionalized micropores with fluorescence microscopy. A.** Schematic illustration of SAPE coupling on PPy-ODN-functionalized micropore. B. Comparison of the transmission and fluorescence images of a micropore. **Figure S2 Fluorescence Activated Cell Sorting (FACS) analysis of the primary splenocyte sample.** The T lymphocytes were labeled by fluorescent R-phycoerythrin conjugated with anti-CD3e and the B lymphocytes were labeled by fluorescent phycoerythrin-Cy7. About 29% and 67% of the cell population are T lymphocytes and B lymphocytes, respectively. **Figure S3 Experimental set-up for cell capture and observation with an inverted transmission microscope.** The chip in PBS buffer was placed on a 1mm-thick plastic support to ensure that non-specific cells could travel across the micropore.
(DOC)

Movie S1 **Real-time cell translocation and capture in an antibody-functionalized micropore.** The cell sample is primary splenocytes containing both T lymphocytes and B lymphocytes. The micropore is functionalized with anti-CD90 IgG specifically targeting T lymphocytes. During their passage along the pore wall, some cells are trapped inside the micropore as a result of specific interactions with antibodies.
(AVI)

Movie S2 **Control experiment of cell translocation through an ODN-modified micropore.** In absence of specific antibodies, all cells pass across the micropore.
(AVI)
