# Supplementary material for: Selective Individual Primary Cell Capture Using Locally Bio-Functionalized Micropores
Source: PLoS One. 2013 Mar 1;8(3):e57717. doi: 10.1371/journal.pone.0057717 (PMC3585871; doi:10.1371/journal.pone.0057717)
Supplement: File S1 — This file contains: Table S1 Sequences of the probes and ODN conjugates used in this study. Anti-CD19 and anti-CD90 are specific to B lymphocytes and T lymphocytes, respectively. Figure S1 Characterization of PPy-ODN-functionalized micropores with fluorescence microscopy. A. Schematic illustration of SAPE coupling on PPy-ODN-functionalized micropore. B. Comparison of the transmission and fluorescence images of a micropore. Figure S2 Fluorescence Activated Cell Sorting (FACS) analysis of the primary splenocyte sample. The T lymphocytes were labeled by fluorescent R-phycoerythrin conjugated with anti-CD3e and the B lymphocytes were labeled by fluorescent phycoerythrin-Cy7. About 29% and 67% of the cell population are T lymphocytes and B lymphocytes, respectively. Figure S3 Experimental set-up for cell capture and observation with an inverted transmission microscope. The chip in PBS buffer was placed on a 1mm-thick plastic support to ensure that non-specific cells could travel across the micropore. (DOC) [file pone.0057717.s001.doc]

**Supporting Information**

**Selective Individual Primary Cell Capture Using Locally Bio-Functionalized Micropores**

Jie Liu, Radoslaw Bombera, Loïc Leroy, Yoann Roupioz, Dieudonné R. Baganizi, Patrice N. Marche, Vincent Haguet, Pascal Mailley, Thierry Livache

**Movie S1** **Real-time cell translocation and capture in an antibody-functionalized micropore.** The cell sample is primary splenocytes containing both T lymphocytes and B lymphocytes. The micropore is functionalized with anti-CD90 IgG specifically targeting T lymphocytes. During their passage along the pore wall, some cells are trapped inside the micropore as a result of specific interactions with antibodies.

**Movie S2** **Control experiment of cell translocation through an ODN-modified micropore.** In absence of specific antibodies, all cells pass across the micropore.

**Supplementary experimental details**

**Text A**

**Reagents and buffers**

The pyrrole-ODN (Py-ODN) conjugates were synthesized following the protocol described by Lassalle *et al*. . Complementary and non-complementary biotinylated ODN (cODN-biot and ncODN-biot) targets, and the intermediate ODN strands (iODN) were purchased from Eurogentec (Angers, France). The Streptavidin-*R*-Phycoerythrin conjugate (SAPE, 1 mg/mL) was supplied by Invitrogen (Cergy-Pontoise, France). Streptavidin-conjugated polystyrene microparticles (10.14±1.04 μm) were purchased from Bangs Laboratories (Fishers, IN, USA). The antibody-conjugated complementary ODN strands (cODN-antibody) were synthesized following the protocol described by Niemeyer *et al*. . All other reagents were purchased from Sigma-Aldrich (France). The ODN sequences are listed in Table S1. The ODN sequences used in this work have a melting temperature (T_m_) higher than 60°C and do not form any secondary structures, which allows performing experiments at room temperature.

Composition of the buffers: *Deposition buffer* (DB): 0.05 M sodium phosphate buffer, 0.05 M NaCl, 10% v/v glycerol, 2% v/v acetonitrile, pH = 6.8. *Hybridization buffer* (HB): 0.02 M sodium phosphate buffer, 1.1 M NaCl, 5.4 mM KCl, 4% v/v 50x Denhardt, 0.2% v/v salmon sperm DNA, 0.3% v/v Tween 20, pH = 7.4. *Washing buffer* (WB): 0.01 M sodium phosphate buffer, 0.55 M NaCl, 2.7 mM KCl, 0.15% v/v Tween 20, pH = 7.4. *PBS buffer*: 0.01 M sodium phosphate buffer, 0.138 M NaCl, 2.7 mM KCl, pH = 7.4.

|  | Name | Sequence |
| --- | --- | --- |
| ODN sequences 1 | Py-ODN | pyrrole-(T)_10_-^5’^GAC CGG TAT GCG ACC TGG TAT GCG^3’^ |
|  | cODN-biot | ^3’^CTG GCC ATA CGC TGG ACC ATA CGC^5’^-biotin |
|  | ncODN-biot | ^3’^ACG CTA CGC TCG CCA TTG GAC TGG^5’^-biotin |
|  | iODN | ^3’^CTG GCC ATA CGC TGG ACC ATA CGC CACGCTCA CTTAAG GCTTCCAT^5’^ |
|  | cODN-anti-CD19 | ^5’^GTGCGAGT GAATTC CGAAGGTA^3’^-(T)_10_-Anti-CD19 |
| ODN sequences 2 | Py-ODN | pyrrole-(T)_10_-^5’^GAC CAT CGT GCG GGT AGG TAG ACC^3’^ |
|  | iODN | ^3’^CTG GTA GCA CGC CCA TCC ATC TGG GAACGGAT GTCGAC TGGAACAG^5’^ |
|  | cODN-anti-CD90 | ^5’^CTTGCCTA CAGCTG ACCTTGTC^3’^-(T)_10_-Anti-CD90 |

**Table S1. Sequences of the probes and ODN conjugates used in this study.** Anti-CD19 and anti-CD90 are specific to B lymphocytes and T lymphocytes, respectively.

**Text B**

**Micropore functionalization with polypyrrole-ODN (PPy-ODN) and fluorescence characterization**

The micropore silicon chips were fabricated as described previously . Before functionalization, the micropore chip was washed with a H_2_O_2_/H_2_SO_4_ (Piranha) solution to remove organic residues and mounted in a homemade mechanical holder. The number of micropores immersed in the electrolyte is adjustable from 1 to 9 so that the pores can be functionalized individually or simultaneously in one step. A deposition buffer containing pyrrole (20 mM) and Py-ODN (5 µM) was introduced into the two electrolyte chambers on each side of the chip. A platinum electrode was placed in each chamber and located at a distance of about 3 mm from the chip surface. A DC voltage pulse of 2 V was applied for 100 ms between the two electrodes to trigger local electropolymerization of pyrrole and pyrrole-ODN on the pore walls. At last, the chip was thoroughly rinsed with deionized water and dried in vacuum.

The PPy-ODN-functionalized micropores were characterized by fluorescence microscopy (Figure S1) . The functionalized chips were first immersed in HB for 5 min at room temperature to block the chip surface and minimize non-specific adsorption. Next, the chips were moved to a 100-nM cODN-biot target solution in HB for 15 min at room temperature for ODN hybridization. Then, the chips were rinsed with WB, incubated in SAPE (0.1 mg/mL in WB) solution for 15 min in dark and rinsed with WB. Finally, the chips were clamped between glass slides to prevent drying during fluorescence microscopy observation. Fluorescence (λ_ex_ = 550 nm, λ_em_ = 590 nm) was recorded using an epifluorescence microscope (BX 60, Olympus) equipped with a Peltier-cooled CCD camera (Hamamatsu) and analyzed with IMAGEPRO PLUS software (Media Cybernetics). The comparison between the transmission and fluorescence images of micropores shows that only the micropore walls are fluorescent (Figure S1B). The presence of fluorescence indicates the presence of deposited PPy-ODN copolymer, which confirms that the observed micropore is locally functionalized.


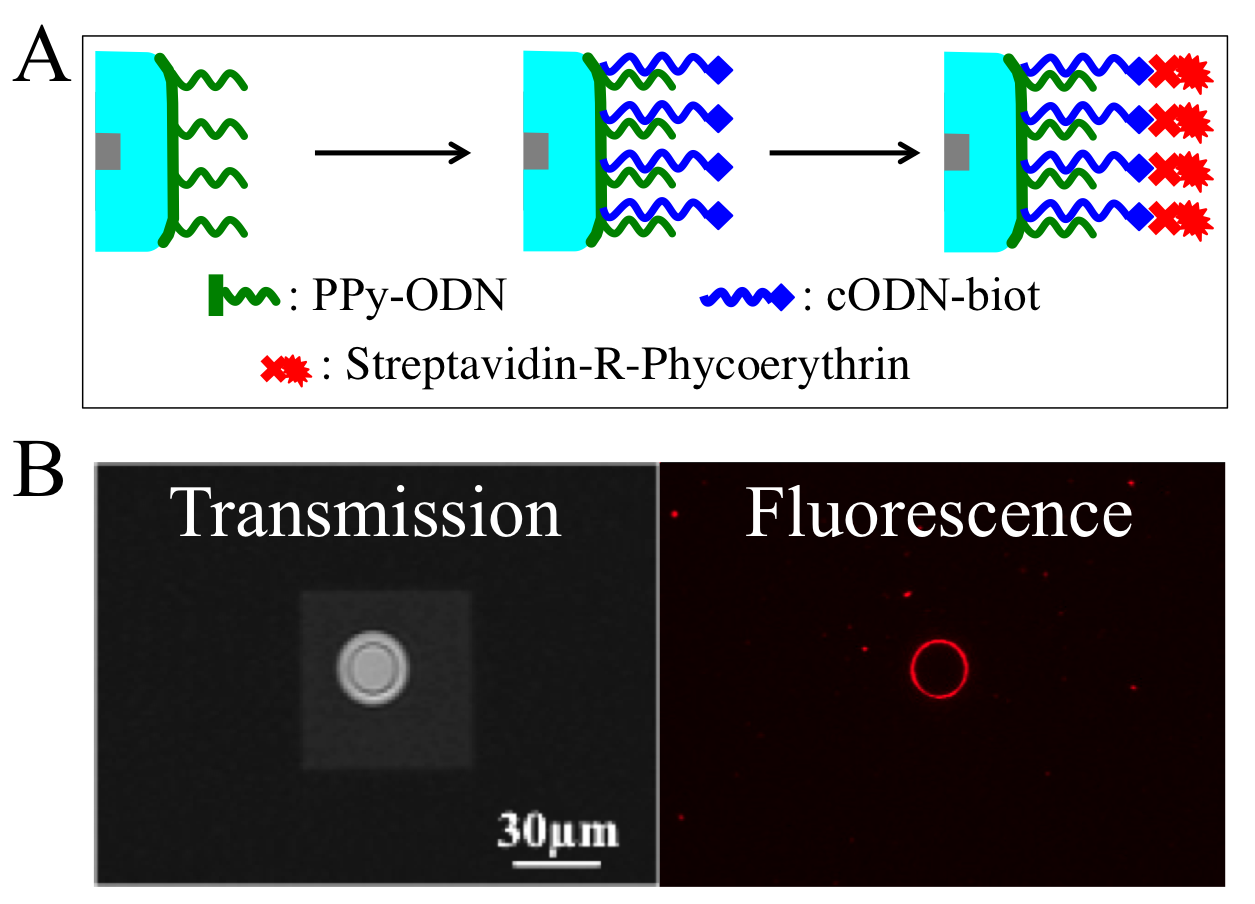


**Figure S1.** **Characterization of PPy-ODN-functionalized micropores with fluorescence microscopy. A.** Schematic illustration of SAPE coupling on PPy-ODN-functionalized micropore. **B.** Comparison of the transmission and fluorescence images of a micropore.


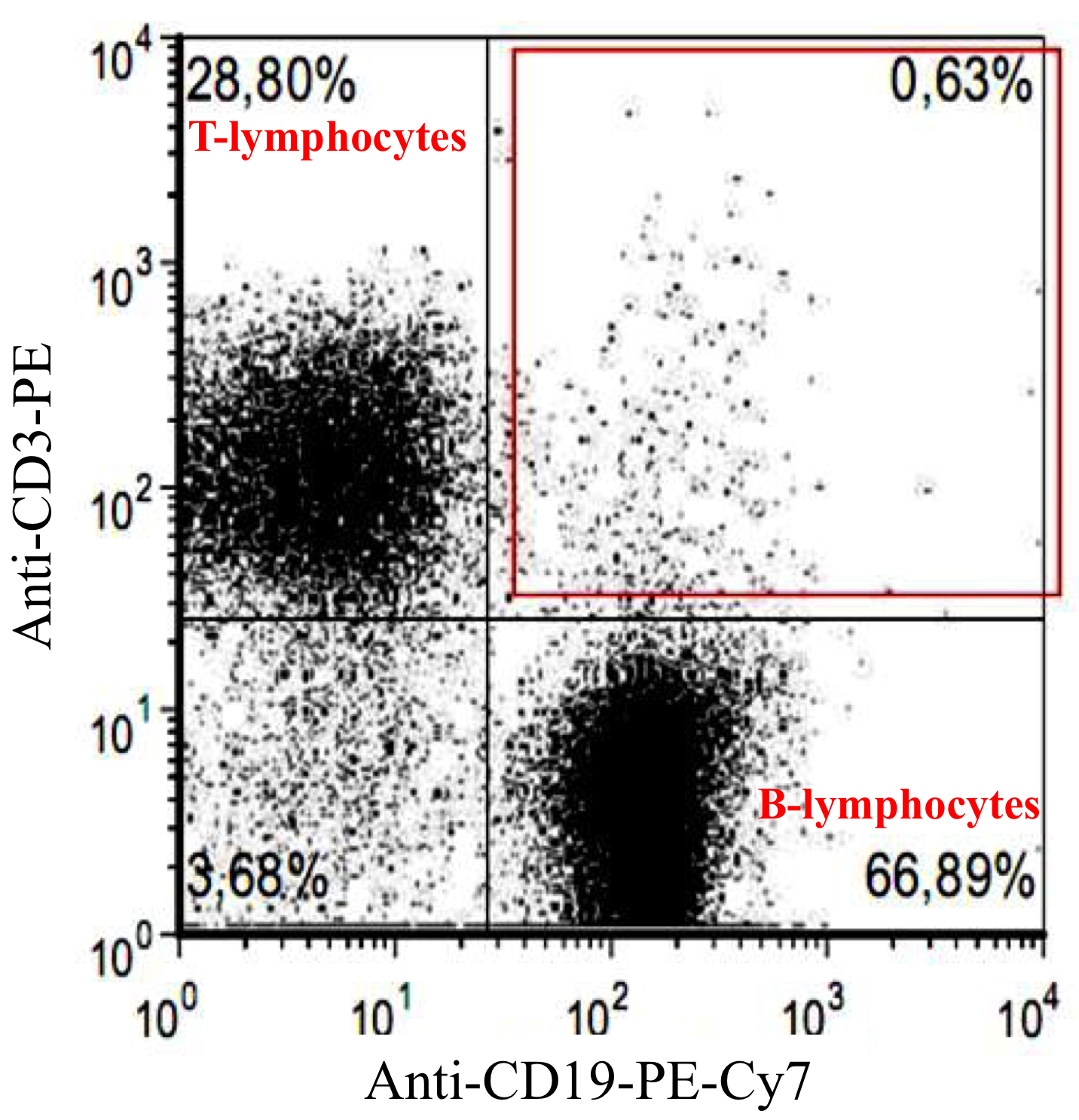


**Figure S2.** **Fluorescence Activated Cell Sorting (FACS) analysis of the primary splenocyte sample.** The T lymphocytes were labeled by fluorescent R-phycoerythrin conjugated with anti-CD3e and the B lymphocytes were labeled by fluorescent phycoerythrin-Cy7. About 29% and 67% of the cell population are T lymphocytes and B lymphocytes, respectively.


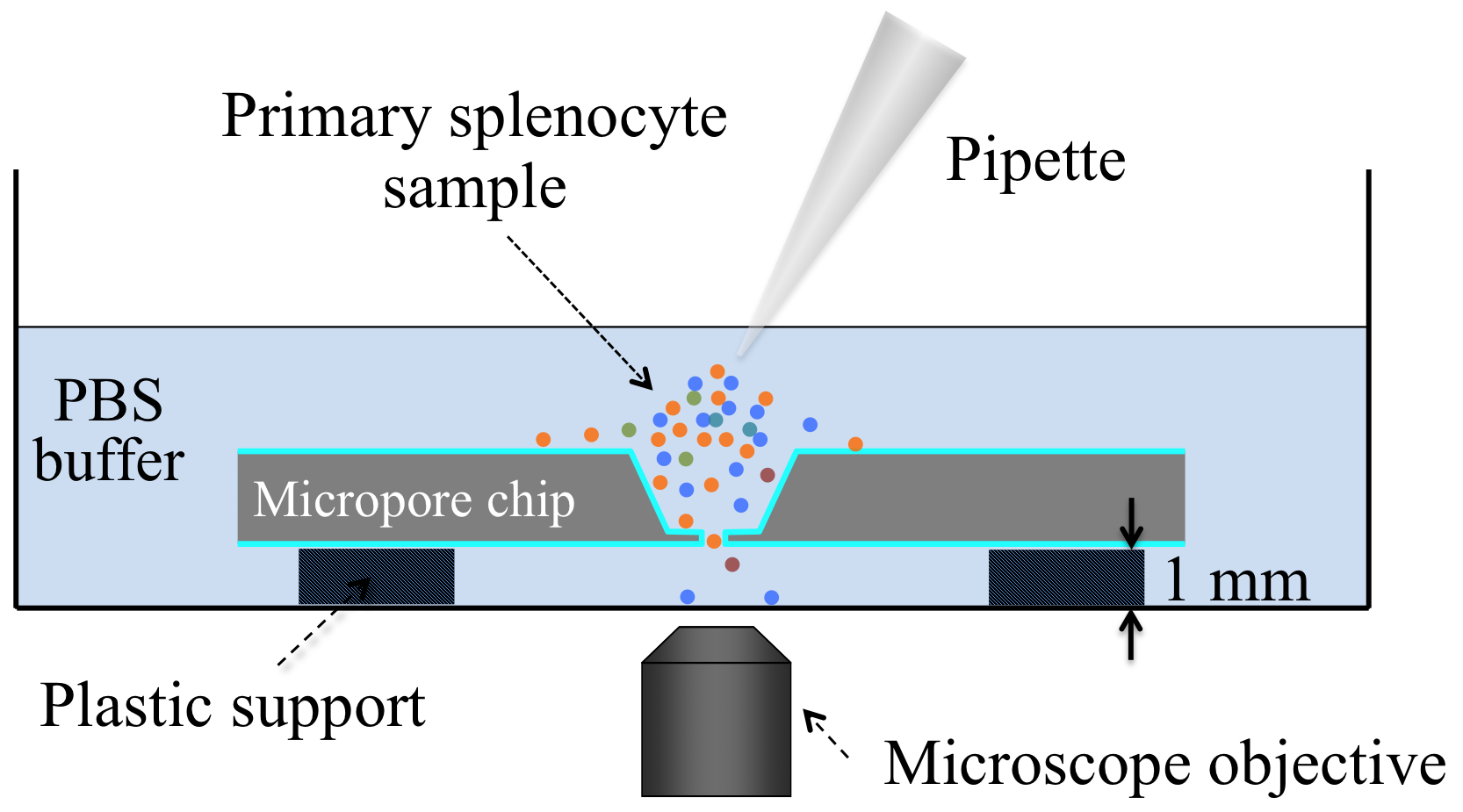


**Figure S3.** **Experimental set-up for cell capture and observation with an inverted transmission microscope.** The chip in PBS buffer was placed on a 1mm-thick plastic support to ensure that non-specific cells could travel across the micropore.

**References:**

1. Lassalle N, Roget A, Livache T, Mailley P, Vieil E (2001) Electropolymerisable pyrrole-oligonucleotide: synthesis and analysis of ODN hybridisation by fluorescence and QCM. Talanta 55: 993-1004.

2. Schroeder H, Ellinger B, Becker CFW, Waldmann H, Niemeyer CM (2007) Generation of Live-Cell Microarrays by Means of DNA-Directed Immobilization of Specific Cell-Surface Ligands. Angew Chem Int Ed 46: 4180-4183.

3. Reisewitz S, Schroeder H, Tort N, Edwards KA, Baeumner AJ, et al. (2010) Capture and Culturing of Living Cells on Microstructured DNA Substrates. Small 6: 2162-2168.

4. Picollet-D’hahan N, Sordel T, Garnier-Raveaud S, Sauter F, Ricoul F, et al. (2004) A Silicon-Based “Multi Patch” Device for Ion Channel Current Sensing. Sensor Lett 2: 91-94.

5. Liu J, Pham P, Haguet V, Sauter-Starace F, Leroy L, et al. (2012) Polarization-Induced Local Pore-Wall Functionalization for Biosensing: From Micropore to Nanopore. Anal Chem 84: 3254-3261.

6. Bouchet A, Descamps E, Mailley P, Livache T, Chatelain F, et al. (2009) Contactless Electrofunctionalization of a Single Pore. Small 5: 2297-2303.
